# Low-sodium diet induces atherogenesis regardless of lowering blood pressure in hypertensive hyperlipidemic mice

**DOI:** 10.1371/journal.pone.0177086

**Published:** 2017-05-08

**Authors:** Fernanda B. Fusco, Diego J. Gomes, Kely C. S. Bispo, Veronica P. Toledo, Denise F. Barbeiro, Vera L. Capelozzi, Luzia N. S. Furukawa, Ana P. P. Velosa, Walcy R. Teodoro, Joel C. Heimann, Eder C. R. Quintao, Marisa Passarelli, Edna R. Nakandakare, Sergio Catanozi

**Affiliations:** 1 Lipids Laboratory (LIM-10) of Endocrinology Division of the University of São Paulo Medical School, São Paulo, SP, Brazil; 2 Department of Pathology of the University of São Paulo Medical School, São Paulo, SP, Brazil; 3 Rheumatology Division (LIM-17) of the University of São Paulo Medical School, São Paulo, SP, Brazil; 4 Emergency Medicine Department (LIM-51) of the University of São Paulo Medical School, São Paulo, SP, Brazil; 5 Experimental Hypertension Laboratory (LIM-16) of the University of São Paulo Medical School, São Paulo, SP, Brazil; University of Basque Country, SPAIN

## Abstract

This study investigated the influence of sodium restriction and antihypertensive drugs on atherogenesis utilizing hypertensive (H) low-density lipoprotein-receptor knockout mice treated or not with losartan (Los) or hydralazine (Hyd) and fed low-sodium (LS) or normal-sodium (NS) chow. Despite reducing the blood pressure (BP) of H-LS mice, the LS diet caused arterial lipid infiltration due to increased plasma total cholesterol (TC) and triglycerides (TG). Los and Hyd reduced the BP of H-LS mice, and Los effectively prevented arterial injury, likely by reducing plasma TG and nonesterified fatty acids. Aortic lipid infiltration was lower in Los-treated H-LS mice (H-LS+Los) than in normotensive (N)-LS and H-LS mice. Aortic angiotensin II type 1 (AT1) receptor content was greater in H-NS than H-LS mice and in H-LS+Hyd than H-LS+Los mice. Carboxymethyl-lysine (CML) and receptor for advanced glycation end products (RAGE) immunostaining was greater in H-LS than H-NS mice. CML and RAGE levels were lower in LS animals treated with antihypertensive drugs, and Hyd enhanced the AT1 receptor level. Hyd also increased the gene expression of F4/80 but not tumor necrosis factor-α, interleukin (IL)-1β, IL-6, IL-10, intercellular adhesion molecule-1 or cluster of differentiation 66. The novelty of the current study is that in a murine model of simultaneous hypertension and hyperlipidemia, the pleiotropic effect of chronic, severe sodium restriction elicited aortic damage even with reduced BP. These negative effects on the arterial wall were reduced by AT1 receptor antagonism, demonstrating the influence of angiotensin II in atherogenesis induced by a severely LS diet.

## Introduction

Observational studies in human populations have shown conflicting results regarding the association of sodium intake with cardiovascular events and mortality [1, 2]. Low-sodium (LS) diets adversely influence markers for cardiovascular disease by chronically activating the renin-angiotensin-aldosterone system (RAAS), increasing the secretion of catecholamines, and worsening the glucose and lipid metabolisms [3]. Despite the hypotensive effects, decreased sodium intake and RAAS activation not only impair insulin sensitivity and glucose tolerance [4] but also lower plasma high-density lipoprotein cholesterol (HDL-C) and apolipoprotein AI levels [5]. In normotensive (N) low-density lipoprotein-receptor knockout (LDLR KO) or apolipoprotein E knockout (apoE KO) mice, chronic LS increases plasma lipid levels, which favors aortic lipid infiltration [6]. In addition to stimulating renal sodium absorption and vasoconstriction, angiotensin II (ANG II) triggers oxidative stress and inflammation, favoring advanced glycation end products (AGE) generation [7].

It is unknown whether a LS-induced blood pressure (BP) reduction in hypertensive (H) animals circumvents the arterial wall lipid infiltration brought about by LS-induced hyperlipidemia. Since dyslipidemia and chronic RAAS activation are atherogenic factors, the present study used hyperlipidemic mice with renovascular hypertension (ANG II-dependent hypertension; 2-kidney, 1-clip—2K1C) as a model to investigate the effects of chronic LS on atherogenesis. In this experimental model, chronic LS elicited vascular damage. Despite its antihypertensive effect, the LS diet led to higher plasma lipid concentrations, enhanced local lipid infiltration, increased carboxymethyl-lysine (CML, an AGE), receptor for advanced glycation end products (RAGE), and tumor necrosis factor (TNF)-α levels and lower angiotensin II type 1(AT1) receptor levels. Overall, these experimental data suggest that long-term dietary sodium restriction brings about undesirable effects on some markers of cardiovascular disease, thus impairing the efficacy of LS in lowering BP for preventing atherosclerotic heart disease and reducing heart disease-related mortality.

## Materials and methods

### Materials

The enzymatic colorimetric kits used for determining the plasma total cholesterol (TC), triglyceride (TG) and nonesterified fatty acid (NEFA) levels were obtained, respectively, from Labtest Brazil, Roche Brazil and Randox Clinical Diagnostic Solutions (Randox of Brazil, Ltda). Twenty-four-hour urinary collection was accomplished in individual metabolic cages obtained from Beiramar Indústria e Comércio, Ltda, São Paulo, SP, Brazil (length x height x width; 30.5 x 35 x 20 cm). Twenty-four-hour urinary sodium (UNa) measurements were performed using an FC 280 flame spectrophotometer (CELM; São Paulo, SP, Brazil). Animal chow, containing either 0.15 or 1.27% sodium chloride, was obtained from Harlan Teklad (Madison, WI, USA). BP was measured with an RTBP 2045 model coupled to an RTBP 001 data acquisition and analysis system (Kent Scientific Corporation, CT, USA) using a Data Acquisition System Laboratory (DASYLab) 7.0 version program (DASYTEC, National Instruments Company, NH, USA).

### Animals

Homozygous LDLR KO mice, inbred on a C57BL/6J background were purchased from Jackson Laboratory (Bar Harbor, ME, USA), and a breeding colony was established in-house. Animals were housed in a conventional animal facility at 22±2°C under a 12-h light/dark cycle and were fed pelleted commercial chow ad libitum (Nuvilab CR1, São Paulo, Brazil) with free access to drinking water. All determinations were made from blood samples (140 μL) drawn from the tail vein into heparinized micro-hematocrit capillary tubes after a 12-h overnight fasting period. Blood samples were centrifuged (3,000 rpm, 4°C, 20 min), and the levels of plasma lipids (TC, TG and NEFA) were then immediately quantified via enzymatic colorimetric methods. Plasma TC and TG levels, hematocrit, and body weight were measured monthly. Animals were placed individually in metabolic cages with free access to food and drinking water for twenty-four-hour UNa assessments. Systolic BP was assessed in conscious mice using a standard tail-cuff technique and an oscillometric method. The mice were mildly warmed up for 30 min prior the BP assessment. Only BP measurements from resting animals were considered. After three successive days of mouse preconditioning to the measurement system, twelve readings were recorded on two consecutive days and averaged to obtain the mean values.

### Renovascular hypertension

Renovascular hypertension (2K1C) was induced according to methods presented in previous studies [8, 9]. Briefly, using a dissecting microscope, rectangular silver clips (width X length; 1.5 mm X 1.0 mm) with rounded edges and a 0.076-mm-wide slit were made. One hour before anesthesia, tramadol chlorhydrate was subcutaneously injected (12.5 mg/kg of body weight) as preemptive analgesia [10]. Ketamine hydrochloride (Ketalar) (100 mg/kg of body weight) and xylazine (Rompun) (10 mg/kg of body weight) were then intraperitoneally injected as anesthesia. The body temperature was maintained within physiological limits using a surgical table heated to 37°C. Celiotomy was performed to place a silver clip around the right renal artery, on the emergence of the renal artery from the aorta [8]. Subcutaneous tramadol chlorhydrate injections (12.5 mg/kg of body weight) were administered every 8 h for 48 h after the surgical procedure. Tramadol chlorhydrate (12.5 mg/kg of body weight) was then administered into the drinking water daily for up to one-week after the 48-h post-surgical period. Enrofloxacin (1 mL/40 kg of body weight) was subcutaneously administered once a day for 3 days. None of the animals became ill or died prior to the experimental endpoint.

### Morphometric studies

After the mice were sacrificed, the heart and thoracic aorta were dissected using a stereoscope for magnification. The mice were then transcardially perfused under low-pressure, first with a cold 0.9% NaCl solution and thereafter with tissue-freezing medium to ensure the proper preservation of histological structures for frozen-tissue specimens. The heart and thoracic aorta were then excised in the fresh state and preserved in liquid nitrogen for future histological evaluation. Cryostat (Leica CM1800 model, MD, USA) serial cross-sections 4 μm in thickness were obtained from an aortic arch segment 10 mm in length, beginning from the aortic root. As previously reported [11, 12], early atherosclerotic lesion development occurs in the inner curvature of the aortic arch and at the beginning of the side branches, which are natural sites of shear stress-related plaque initiation. Variations in the blood flow patterns in different vascular regions have complex effects on the development of atherosclerosis. With the aim of facilitating the time-consuming vascular cross-sectional analysis, four aortic arch segments (named segments I, II, III and IV; each 140 μm long) with 1-mm intervals between them were investigated. Aortic arch segments III and IV were located closer to the heart than were segments I and II. Three sets of 10 cryostat cross-sections (each 4-μm thick), at 100-μm intervals between each set, were serially obtained from segments I, II, III and IV, yielding 120 aortic cross-section samples from each animal. Fifteen cross-sections of each segment were randomly chosen for histological analysis. Thus, sixty samples of the aortic arch of each animal were microscopically analyzed. Alternate sections of each segment were used for the lipid content and immunofluorescence analyses. To analyze lipid infiltration into the aortic arch wall, samples were stained with oil red O and counterstained with hematoxylin, according to the modified method reported by Paigen *et al* [13].

### Immunofluorescence assays

The AT1 receptor, CML and RAGE contents in the aortic arch wall were evaluated by immunofluorescence. Specimens were mounted on glass slides with 3-aminopropyltriethoxysilane (Sigma Chemical, Co.), washed in phosphate-buffered saline (PBS) and blocked with 5% bovine serum albumin in PBS for 30 min at room temperature. The slices were incubated overnight at 4°C with rabbit polyclonal anti-AT1 receptor (1:40; Santa Cruz Biotechnology, Inc., Cat # SC1173, Dallas, TX, USA), rabbit polyclonal anti-CML (1:40; Immundiagnostik AG, Cat # 1109, Bensheim, Germany) and rabbit polyclonal anti-RAGE (1:50; Santa Cruz Biotechnology, Inc., Cat # SC 5663, Dallas, TX, USA) antibodies diluted in PBS. The sections were next washed in PBS with 0.05% Tween-20 and incubated for 90 min at room temperature with Alexa 488-conjugated goat anti-rabbit IgG antibody (Life Technologies, Cat # A11008, Grand Island, NY, USA) diluted at 1:200 in a PBS solution containing 0.006% Evans blue dye. Finally, the samples were mounted in buffered glycerol and analyzed using fluorescence microscopy (Olympus BX51, Olympus, Co., Tokyo, Japan). For negative and autofluorescence controls, sections were incubated with PBS instead of the specific antibody.

### Histomorphometric quantification

The extent of lipid infiltration and the AT1 receptor, CML and RAGE contents in the subendothelial space and media layer of the aortic arch tissue were evaluated in each histological section utilizing an image analysis system by an independent and experienced field investigator blinded to the study protocol. Briefly, the equipment consisted of an Olympus camera (Olympus, Co., St. Laurent, Quebec, Canada) coupled to an Olympus microscope (Olympus BX51, Olympus, Co., Tokyo, Japan), from which the images were sent to an LG monitor via a digitizing system (Oculus TCX, Coreco, Inc., St. Laurent, Quebec, Canada) and downloaded to a computer (Pentium 1330 MHz). The images were then processed with Image-Pro-Plus 6.0 software. To quantify lipid infiltration, the CML content and the AT1 receptor and RAGE expression levels in the cross-sectional areas, digital images of either positively stained or immunostained sections were obtained at the level of the aortic arch (from the intima to the external elastic lamina). Care was taken to exclude normal-appearing medial tissue and to include only injured areas of the intima and media layers. Data are expressed as the mean percentage of the total positively stained area of the aortic arch cross-sections.

### Real-time PCR (RT-PCR)

Total RNA was extracted from the aortic arch samples using 1 mL of TRIzol reagent, as described by the manufacturer (Invitrogen, USA). After incubation for 5 min at room temperature, chloroform (200 μL) was added to the tubes and centrifuged at 12,000 × g. The aqueous phase was transferred to another tube, and then the RNA was pelleted by centrifugation (12,000 × g) with cold ethanol and dried in open air. RNA pellets were eluted in RNase-free water and treated with DNase I (Invitrogen, USA). Samples of total RNA were quantified by measuring the optical density at 260 and 280 nm (Nano Vue Plus-spectrophotometer GE, NJ, USA). The integrity of the RNA was assessed by the 260/280 nm ratio and by electrophoresis using a 1% agarose gel stained with ethidium bromide.

The expression levels of AT1 receptor (*Agtr1*), RAGE (*Ager*) and the inflammatory mediators TNF-α (*Tnf*), interleukin (IL)-6 (*Il6*), IL-10 (*Il10*), IL-1β (*Il1β*), macrophage biomarker cluster of differentiation 66 (CD66; *Hepacam2*), F4/80 (*Adgre1*), intracellular adhesion molecule 1 (ICAM-1; *Icam1*) and vascular cell adhesion molecule 1 (VCAM-1; *Vcam1*) were evaluated by RT-PCR. All RT-PCR reaction mixtures were prepared using Superscript Platinum III one-step kits with SYBR Green incorporated (Invitrogen, Cat # 11736–051, USA). cDNA production and DNA amplification were performed on a Step One (Applied Biosystems Carlsbad, CA, USA) thermocycler with 100 ng of total RNA per sample. All products were confirmed by size on a 1.5% agarose gel. Relative expression was determined by the 2^-ΔΔCT^ method using the housekeeping gene β-2 microglobulin (β2M) for normalization. The primer sequences used for this protocol are shown in **Table 1**.

**Table 1:** Gene-specific RT-PCR primers.

*Gene*	Forward	Reverse	Amplicon (bp)
*β2M*	CATGGCTCGCTCGGTGACC	AATGTGAGGCGGGTGGAACTG	148
*Tnf*	CGGCATGGATCTCAAAGACAAC	AAATCGGCTGACGGTGTGG	130
*Il1β*	CAGGCAGGCAGTATCACTCA	AGCTCATATGGGTCCGACAG	248
*Il6*	GGGACTGATGCTGGTGACAACC	AAGCCTCCGACTTGTGAAGTGG	116
*Il10*	TGCCAAGCCTTATCGGAAATG	AAATCGATGACAGCGCCTCAG	149
*Icam1*	CGAAGGTGGTTCTTCTGAGC	GTCTGCTGAGACCCCTCTTG	237
*Vcam1*	ATTTCTGGGGCAGGAAGTT	ACGTCAGAACAACCGAATCC	238
*Adgre1*	CCTGAACATGCAACCTGCCAC	GGGCATGAGCAGCTGTAGGATC	202
*Hepacam2*	TACACTGTCCACGGCATCAGG	CATCATGGTGAACTTGTGCTGG	190
*Agtr1*	CCCTGGCAAGCATCTTATGT	CCAGCAGACCACTGAGCATA	161
*Ager*	GAAGGCTCTGTGGGTGAGTC	TCCGCTTCCTCTGACTGATT	197

Reverse transcription was carried out at 50°C for 10 min and 95°C for 5 min. After reverse transcription, RT-PCR was performed using the following conditions: 95°C for 30 s, 60°C for 30 s and 72°C for 30 s for 35 cycles [14].

### Experimental protocol

This study was approved by the Institutional Animal Care and Research Advisory Committee (CAPPesq HC-USP # 341/10—Comissão de Ética para Análise de Projetos de Pesquisa do Hospital das Clínicas da Faculdade de Medicina da USP), and was strictly performed according to the U.S. National Institutes of Health Guide for the Care and Use of Laboratory Animals [15].

Newly weaned 3-week-old male LDLR KO mice were fed ad libitum pelleted chow containing the following nutrients (g/100 g): casein (28.7), sucrose (31.3), corn starch (20.0), soybean oil (6.0), minerals and vitamins. Increasing amounts of cellulose were replaced by sodium chloride. Mice were randomly assigned to experimental groups according to different sodium chloride concentrations in the diet and/or antihypertensive drug treatments. Mice were fed either a LS diet containing 0.15% sodium chloride or chow containing a standard sodium concentration (NaCl 1.27%), i.e., a NS diet. The LS groups were fed the minimum amount of sodium chloride required for a normal rodent growth rate [16]. Individual animal body weight and group chow intake follow-up started after weaning, and these parameters were monitored three times per week until the end of the experiment. At 2.5 months of age, a silver clip was placed around the right renal artery of animals assigned to the H group. Mice fed the LS diet were redistributed into groups that received drinking water containing either losartan (Los), an AT1 receptor blocker (20 mg/kg of body weight/day), or hydralazine (Hyd), a vasodilator with a direct effect on vascular smooth muscle (15 mg/kg of body weight/day) [17]. Mice fed either LS or NS chow neither having a clip on the renal artery nor receiving an antihypertensive treatment were labeled normotensive (N) mice. Due to photosensitivity, Los was offered in the drinking water in dark bottles and replaced on alternate days. One month after clip placement on the renal artery, mouse BP was monitored. At 5 months of age, animals were placed in individual metabolic cages for 24-hour UNa assessments. Body weight, hematocrit, BP and plasma TC, TG and NEFA measurements were performed at the end of the experiment. The aortic arches from some mice were used for lipid infiltration and immunofluorescence assays, while those of other mice were utilized for gene expression assessments, considering that all animals were simultaneously subjected to the same experimental conditions.

**Fig 1:**
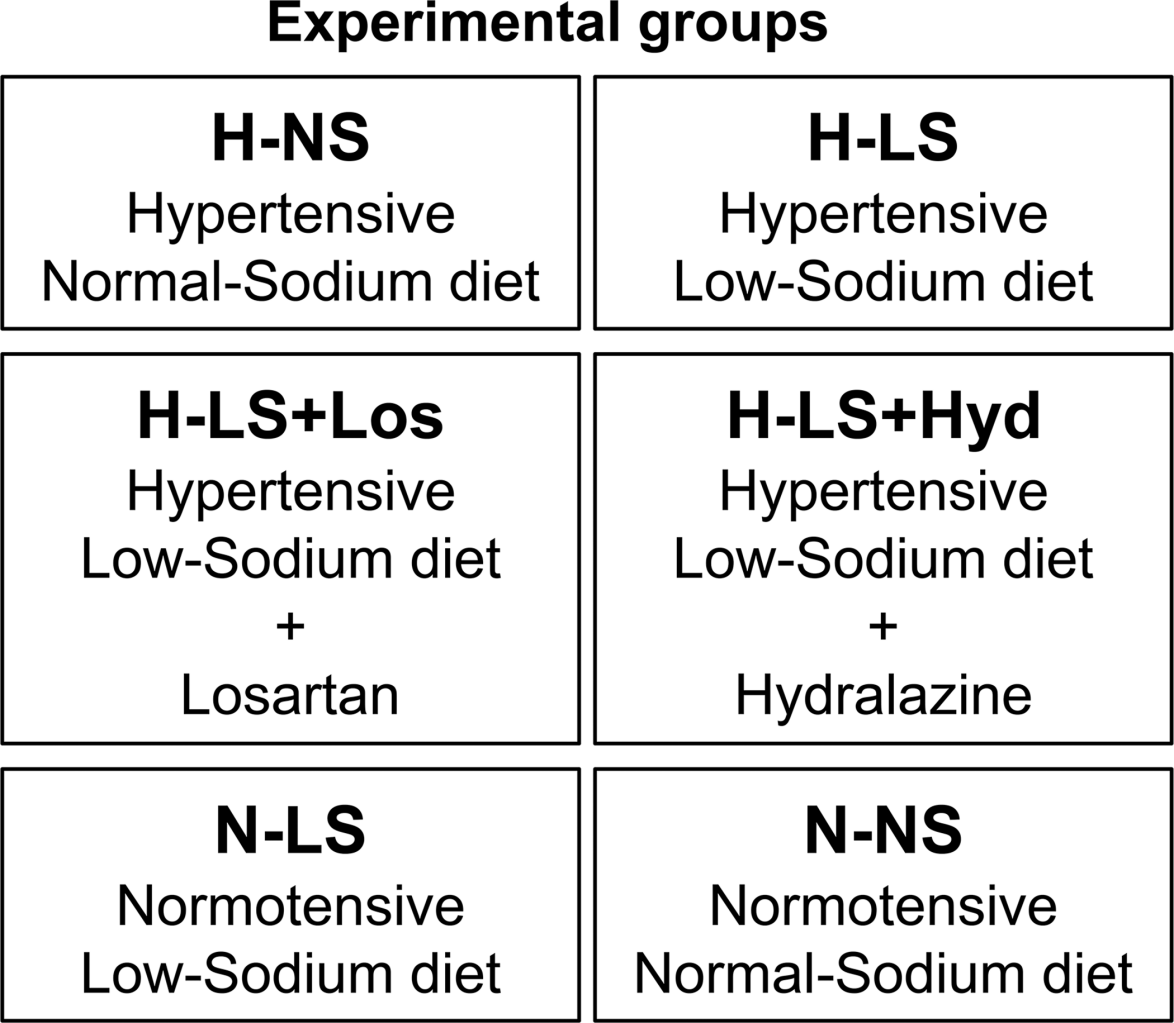
Experimental groups.

Six experimental groups were established (**Fig 1**): the hypertensive normal- sodium (H-NS) group, containing-animals with a clip on the renal artery and fed a normal-sodium diet; the hypertensive low-sodium (H-LS) group, containing-animals with clip a on the renal artery and fed a low-sodium diet; the hypertensive low-sodium + losartan (H-LS+Los) group, containing-animals with clip a on the renal artery, fed a low-sodium diet and treated with losartan; the hypertensive low-sodium + hydralazine (H-LS+Hyd) group, containing-animals with a clip on the renal artery, fed a low-sodium diet and treated with hydralazine; the normotensive low-sodium (N-LS) group, containing-animals without a clip on the renal artery and fed a low-sodium diet; and the normotensive normal-sodium (N-NS) group, containing-animals without a clip on the renal artery and fed a normal-sodium diet.

### Statistical analyses

Statistical comparisons were performed using GraphPad Prism 5 (GraphPad Software, Inc., San Diego, CA, 2007). Unpaired Student t tests or Mann Whitney tests were used for comparing the N-NS with the N-LS groups and the H-NS with the H-LS groups. Kruskal Wallis one-way ANOVA by ranks followed by Dunn’s test (to correct for multiple comparisons) was applied for comparisons among LS groups. Univariate correlations to examine the potential effects of BP and plasma TC, TG, and NEFA concentrations on arterial wall lipid accumulation were performed by Spearman's analysis. Data are expressed as the mean ± standard deviation (SD) or the median (range) according to the parametric or nonparametric analysis used, respectively. The significance levels was at *P* < 0.05.

## Results

### Sodium restriction effects on the physiological parameters of LDLR KO mice

Reduced dietary sodium intake has been a recommended approach for preventing and treating cardiovascular and renal diseases [18, 19]. However, it has been shown that reducing sodium intake may adversely affect hormone and plasma lipid levels, mitigating its hypotensive benefit [20]. Therefore, in the current study, we evaluated the effects of dietary sodium restriction on atherogenesis in hyperlipidemic H mice. Either N or 2K1C LDLR KO mice were assigned to experimental groups according to the different sodium chloride contents in the chow and/or the antihypertensive drug treatment provided. BP and renal weight were measured to ascertain the establishment of the renovascular hypertension model. Possible influences of dietary sodium restriction on body weight, hematocrit, 24-hour UNa excretion and plasma lipid concentrations were also assessed. The LS diet did not affect body weight or hematocrit in N or H mice (**Table 2**). Treatment with Los, an AT1 receptor blocker, allowed the investigation of the contribution of the AT1 receptor signaling pathway on experimental atherogenesis. Such effects were compared with those of treatment with Hyd, which has antihypertensive efficacy independent of AT1 receptor blockage. The hematocrit in the H-LS+Los group was slightly reduced compared with that of the other LS groups (**Table 2**). UNa measurements confirmed the long-term dietary sodium restriction to which the animals were subjected. As expected, N and H animals on a chronic LS diet showed decreased 24-hour UNa excretion and BP compared with those of animals on the NS diet (**Table 2**). The BP of H-LS+Los and H-LS+Hyd mice was lower than of H-LS mice and similar to that of N-LS mice (**Table 2**). Likewise, in other studies [20–24], including ours [6, 25, 26], the LS diet raised plasma lipid concentrations. Accordingly, in the present study, N-LS and H-LS mice developed higher plasma TC and TG concentrations than did mice on the NS diet (**Table 2**). The plasma NEFA concentration was not different between the NS and LS groups (**Table 2**). Among the LS groups, plasma TC did not differ (**Table 2**); however, TG and NEFA were reduced by the Los treatment (H-LS+Los) (**Table 2**).

**Table 2:** Phenotypic characteristics of normotensive (N) or hypertensive (H) LDLR KO mice fed either a normal-sodium (NS) or a low-sodium (LS) diet and treated or not with losartan (Los) or hydralazine (Hyd).

	N-NS (n = 7)	H-NS (n = 8)	N-LS (n = 8)	H-LS (n = 8)	H-LS + Los (n = 7)	H-LS + Hyd (n = 8)
Body weight (g)	24.5 ± 0.6	24.7 ± 1.7	22.6 ± 0.9	22.8 ± 2.5	21.2 ± 2.5	24 ± 0.5
Hematocrit (fraction)	0.51 ± 0.2	0.52 ± 0.3	0.53 ± 0.4	0.53 ± 0.2	0.45 ± 0.4 ^c^	0.48 ± 0.3
BP (mmHg)	76 ± 3	131 ± 4	67 ± 7 ^a^	107 ± 3 ^b^^,^ ^c^	59 ± 4	65 ± 6
UNa (mEq/24 h)	0.043 ± 0.015	0.077 ± 0.047	0.024 ± 0.008 ^a^	0.027 ± 0.018 ^b^	0.027 ± 0.008	0.020 ± 0.009
TC (mmol/L)	13.1 ± 0.8	14.1 ± 1.5	16.6 ± 2.8 ^a^	18.6 ± 4.9 ^b^	19.7 ± 2.6	15.1 ± 2.77
TG (mmol/L)	1.86± 0.17	1.42 ± 0.81	2.99 ± 0.75 ^a^	2.53 ± 0.38 ^b^	1.64 ± 1.05 ^c^	2.72 ± 0.82
NEFA (mmol/L)	1.97 ± 0.17	1.38 ± 0.27	1.64 ± 0.77	1.74 ± 0.47	1.01 ± 0.26 ^c^	1.84 ± 0.52

Number of animals (n); mean ± SD.

^a^ *P* < 0.05 N-NS *vs* N-LS, unpaired Student's t test

^b^ *P* < 0.05 H-NS *vs* H-LS

unpaired Student's t test

^c^ *P* < 0.05 comparing all LS groups, ANOVA with Newman Keuls post hoc test.

Renal mass was assessed to show that the clip effectively constricted the renal artery and reduced renal perfusion [8, 9]. Mice with a clip around the right renal artery showed lower absolute right renal weight and lower renal weight relative to body weight compared with the left kidney (**Table 3**).

**Table 3:** Absolute renal weight and renal weight relative to body weight in either normotensive (N) or hypertensive (H) LDLR KO mice fed either a normal-sodium (NS) or a low-sodium (LS) diet and treated or not with an antihypertensive drug.

Group	Right Kidney		Left Kidney	
	Absolute (mg)	Relative (mg/g)	Absolute (mg)	Relative (mg/g)
H-NS (n = 8)	60 ± 50 ^a^	2.56 ± 2.25 ^b^	210 ± 30	8.41 ± 1.16
H-LS (n = 8)	20 ± 10 ^a^	1.04 ± 0.41 ^b^	200 ± 60	8.63 ± 1.78
H-LS+Los (n = 7)	20 ± 10 ^a^	1.16 ± 0.27 ^b^	190 ± 30	8.87 ± 0.63
H-LS+Hyd (n = 8)	30 ± 5 ^a^	1.15 ± 0.20 ^b^	200 ± 20	8.29 ± 1.03
N-LS (n = 8)	150 ± 30	6.63 ± 0.85	150 ± 30	6.53 ± 1.04
N-NS (n = 7)	170 ± 20	6.76 ± 0.67	160 ± 20	6.71 ± 0.72

Five-month-old male LDLR KO mice, without (N) or with a clip on the right renal artery (renovascular hypertension–H) fed either NS or LS chow ad libitum for 130 days. H-LS animals were treated for 2.5 months with either losartan (Los) or hydralazine (Hyd) in their drinking water. The results are expressed as the mean ± SD; number of animals (n). Unpaired Student's t tests were used for comparisons between groups.

^a^ *P* < 0.0001 (absolute values)

^b ^*P* < 0.0001 (relative values) right kidney *vs* left kidney. Renal relative weight: (mg of renal weight/g of body weight).

### Sodium restriction effects on aortic injury

**Fig 2:**
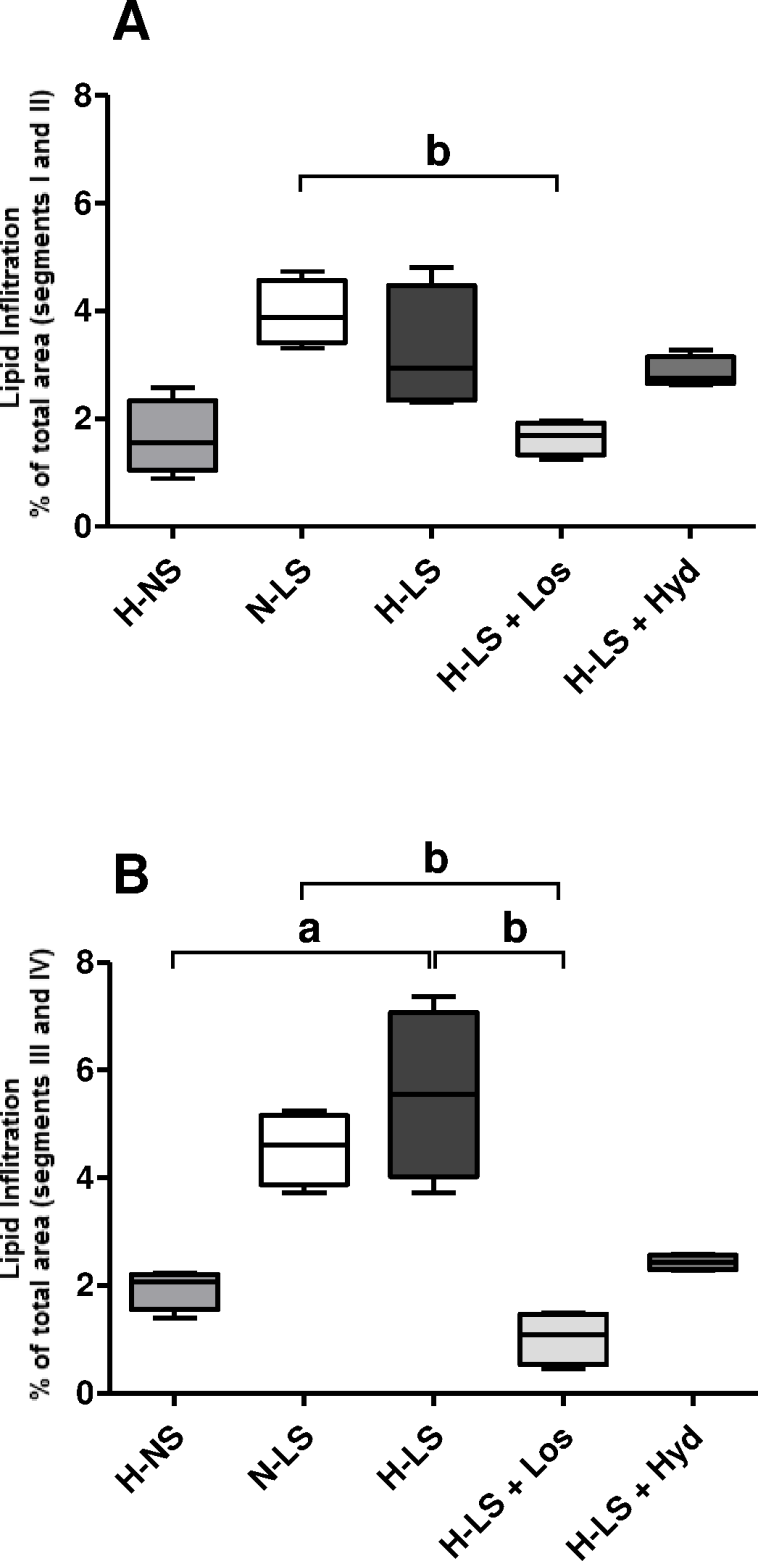
Vascular injury quantified by a histomorphometric analysis of oil red O-stained lipid infiltration as the mean percentage of total positively stained area of the aortic arch cross-sections (**A** segments I and II; **B** segments III and IV); n = 4 mice per group. ^a^ *P* < 0.05, hypertensive mice fed a normal-sodium diet (H-NS) *vs* hypertensive mice fed a low-sodium diet (H-LS), Mann Whitney test. ^b^ *P* < 0.05, Kruskal Wallis with Dunn’s post hoc test applied for comparisons among LS groups.

**Fig 3:**
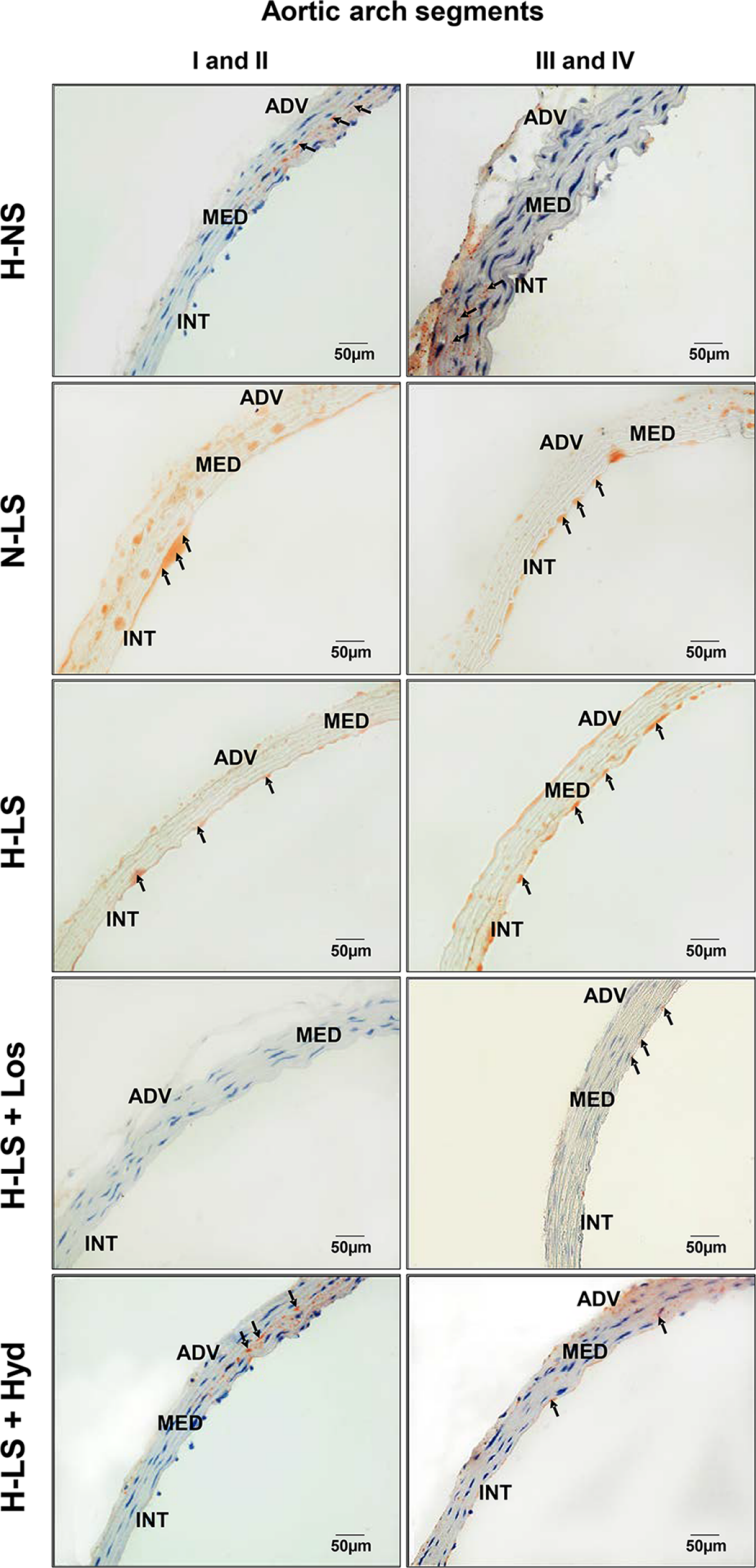
Representative examples of lipid infiltration visualized by oil red O staining of the intima and media layers of different aortic arch segments from animal groups consisting of hypertensive mice fed a normal-sodium diet (H-NS), normotensive mice fed a low-sodium diet (N-LS), hypertensive mice fed a low-sodium diet (H-LS), hypertensive mice fed a low-sodium diet and treated with losartan (H-LS+Los) and hypertensive mice fed a low-sodium diet and treated with hydralazine (H-LS+Hyd). Arrows indicate sites of aortic arch lipid infiltration. ADV = adventitia, MED = media, INT = intima (400×).

To investigate the effects of dietary sodium restriction on arterial damage in the context of simultaneous hyperlipidemia and ANG II-dependent hypertension, five-month-old male N or H LDLR KO mice were sacrificed for the assessment of injury to the aortic arch, a site where atherosclerotic lesions commonly develop in mice [27–29]. Lipid infiltration into the intima and media layers of distal segments (I-II) of the aortic arch was not different between the H-NS and H-LS groups (**Fig 2A**); however, in segments III-IV, lipid infiltration was higher in H-LS than in H-NS mice (**Figs 2B and 3**). When comparing all LS groups, lipid infiltration was lower in the H-LS+Los group than in the N-LS group (aortic segments I, II, III and IV; **Figs 2A, 2B and 3**) and in the H-LS group (aortic segments III and IV; **Figs 2B and 3**). The univariate analysis showed that only the plasma TG and NEFA concentrations were related to the aortic arch lipid infiltration area (**Table 4**).

**Table 4:** Univariate regression analysis of arterial lipid infiltration in N and H LDLR KO mice fed a NS or LS diet treated or not with an antihypertensive drug.

Variables	Segments I, II		Segments III, IV	
	*r*	*P*	*r*	*P*
TC	0.005	0.982	0.245	0.299
TG	**0.688**	**0.001**	**0.582**	**0.007**
NEFA	0.358	0.122	**0.478**	**0.033**
BP	- 0.124	0.604	0.176	0.457

Spearman’s correlations were evaluated considering positive lipid staining areas inside the arterial wall of the aortic arch and BP, as well as plasma TC, TG and NEFA concentrations; n = 20.

**Fig 4:**
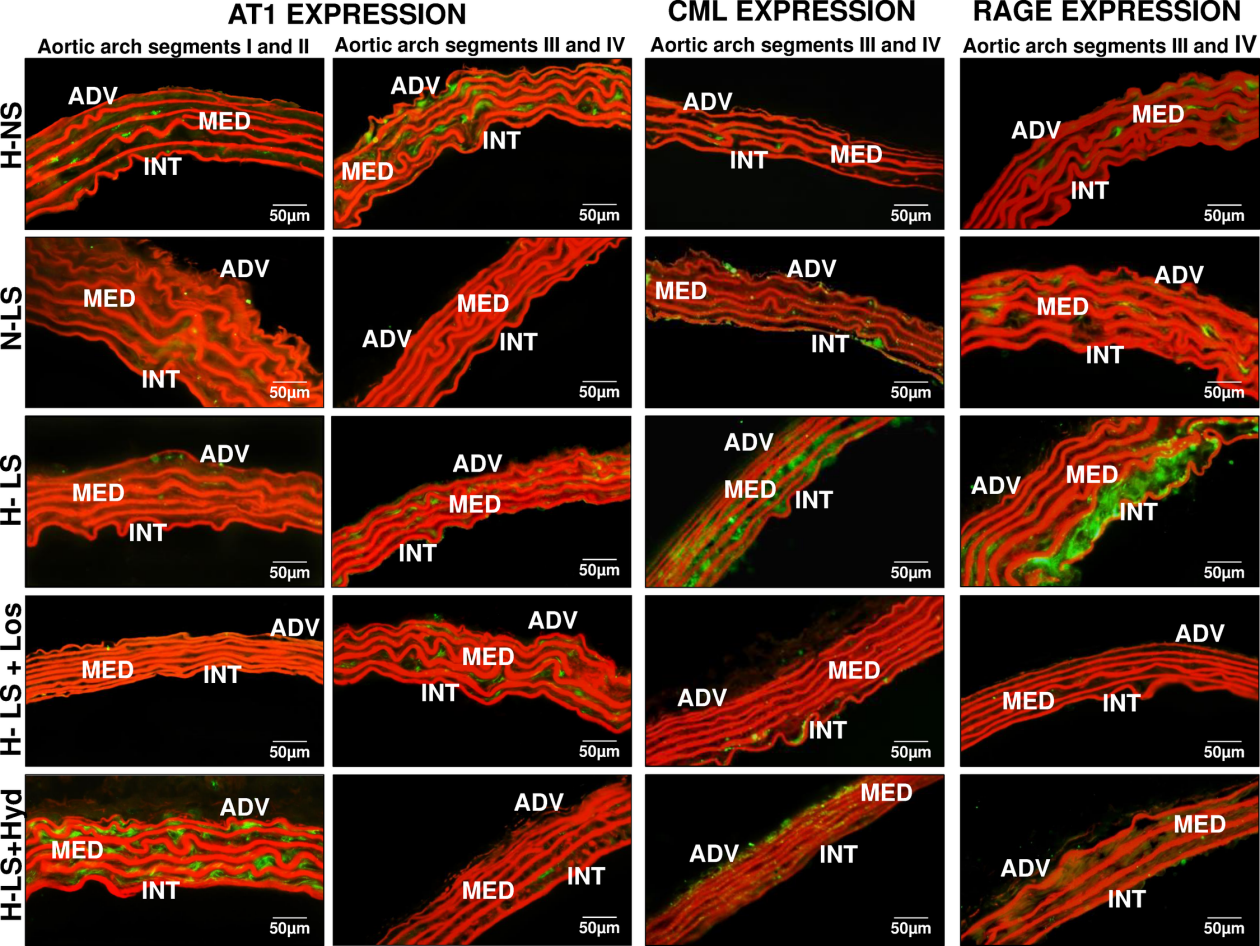
Representative examples of AT1 receptor expression, CML content and RAGE expression visualized by immunofluorescence in the intima and media layers of different aortic arch segments from animal groups consisting of hypertensive mice fed a normal-sodium diet (H-NS), normotensive mice fed a low-sodium diet (N-LS), hypertensive mice fed a low-sodium diet (H-LS), hypertensive mice fed a low-sodium diet and treated with losartan (H-LS+Los) and hypertensive mice fed a low-sodium diet and treated with hydralazine (H-LS+Hyd). ADV = adventitia, MED = media, INT = intima (400×).

**Fig 5:**
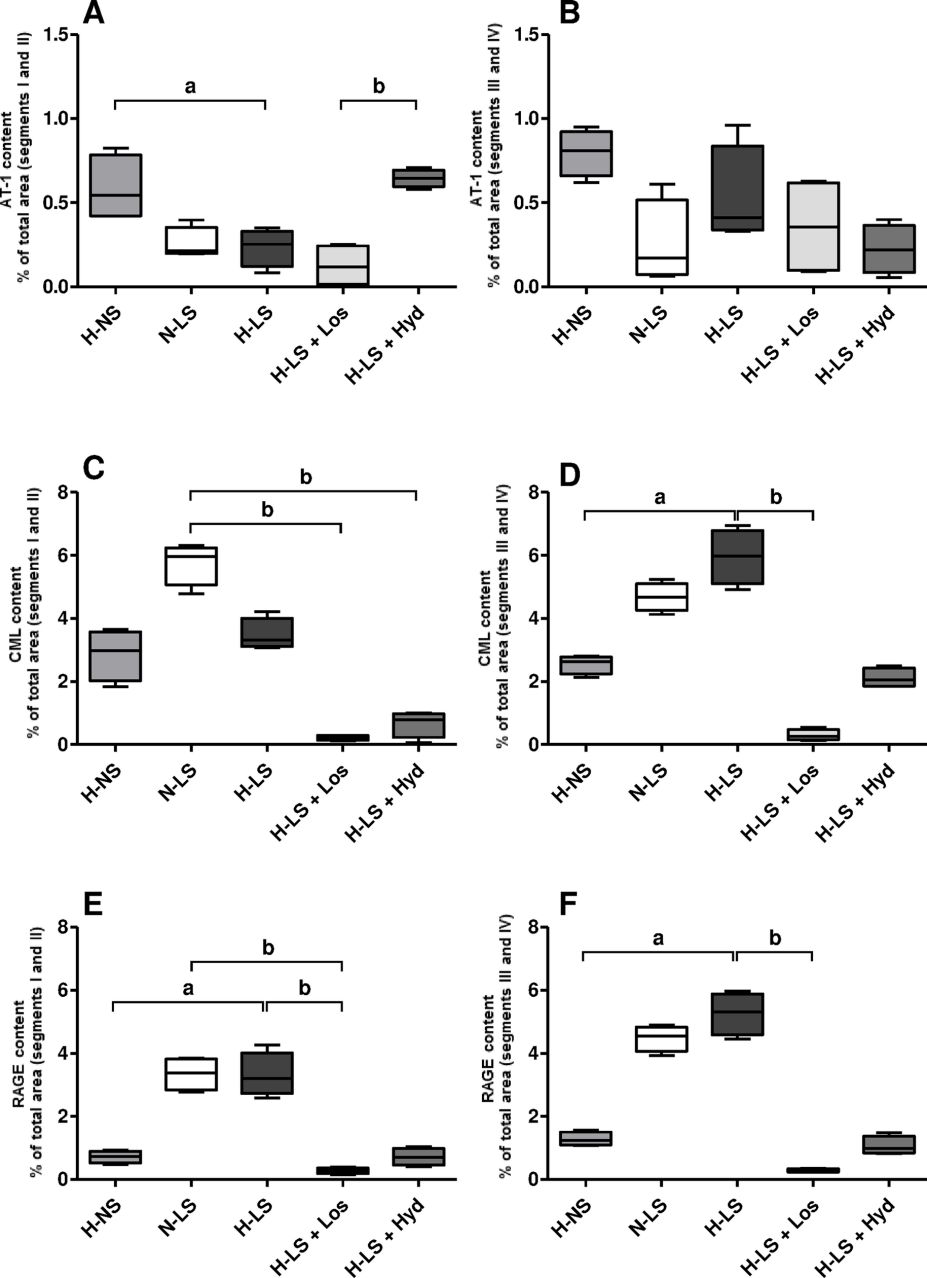
Histomorphometric analysis of immunofluorescence-stained AT1 receptor (**A** segments I and II; **B** segments III and IV), and vascular injury quantified by a histomorphometric analysis of immunofluorescence-stained CML (**C** segments I and II; **D** segments III and IV) and RAGE (**E** segments I and II; **F** segments III and IV); data are represented as the mean percentage of the total positively stained area of the aortic arch cross-sections; n = 4 mice per group. ^a^ *P* < 0.05, hypertensive mice fed a normal-sodium diet (H-NS) *vs* hypertensive mice fed a low-sodium diet (H-LS), Mann Whitney test. ^b^ *P* < 0.05, Kruskal Wallis with Dunn’s post hoc test applied for comparisons among LS groups.

Furthermore, the effect of long-term RAAS activity on vascular AT1 receptor expression was shown by immunofluorescence staining in aortic arch segments I-II, indicating higher AT1 receptor content in the H-NS group than in the H-LS group (**Figs 4 and 5A**). Comparing all LS groups, AT1 receptor immunofluorescence staining in aortic arch segments I-II was higher in Hyd-treated mice than Los-treated mice (**Figs 4 and 5A**). AT1 receptor staining was not different in aortic segments III-IV among the groups (**Figs 4 and 5B**).

Since lipid peroxidation is an important source of AGE biogenesis [7], whether chronic LS had any influence on aortic CML and RAGE contents or on their contribution to atherogenesis was investigated. CML was not different in arterial segments I-II of the H-NS and H-LS groups (**Figs 4 and 5C**). However, CML-positive staining was greater in aortic arch segments III-IV of the H-LS group than the H-NS group (**Figs 4 and 5D**). Mice treated with either Los or Hyd had lower CML content in aortic arch segments I-II than did N-LS mice (**Figs 4 and 5C**). The CML level was enhanced only in arterial segments III-IV of H-LS mice compared with H-NS and Los-treated mice (**Figs 4 and 5D**). Immunofluorescence staining showed that RAGE was increased in all the investigated aortic segments of H-LS mice compared with H-NS mice (**Figs 4, 5E and 5F**). Similarly, the CML and RAGE levels were lower in all aortic segments of H-LS+Los mice (**Figs 4, 5E and 5F**).

### Influence of LS diet and antihypertensive agents on vascular gene expression levels of the AT1 receptor, RAGE and inflammatory mediators

**Fig 6:**
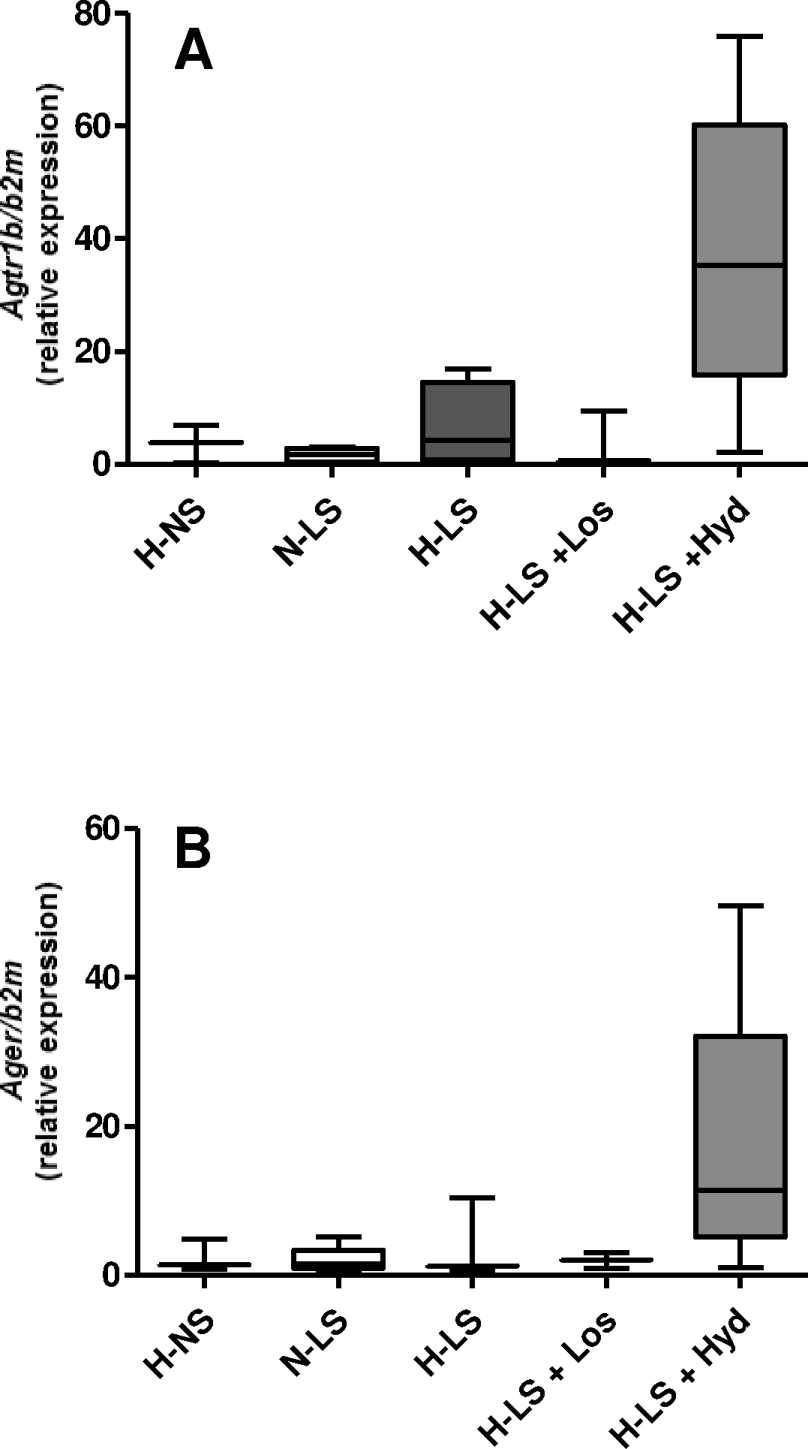
Gene expression (mRNA) of the AT1 receptor (*Agtr1*) and RAGE (*Ager*) in the mouse aortic arch. Data are expressed as relative mRNA units normalized to mouse β2M expression. Mann Whitney test was used for comparisons between hypertensive mice fed a normal-sodium (H-NS) diet and hypertensive mice fed a low-sodium (H-LS) diet. The Kruskal Wallis test with Dunn’s post hoc test was applied for comparisons among the LS groups; n ≥ 4 mice per group.

**Fig 7:**
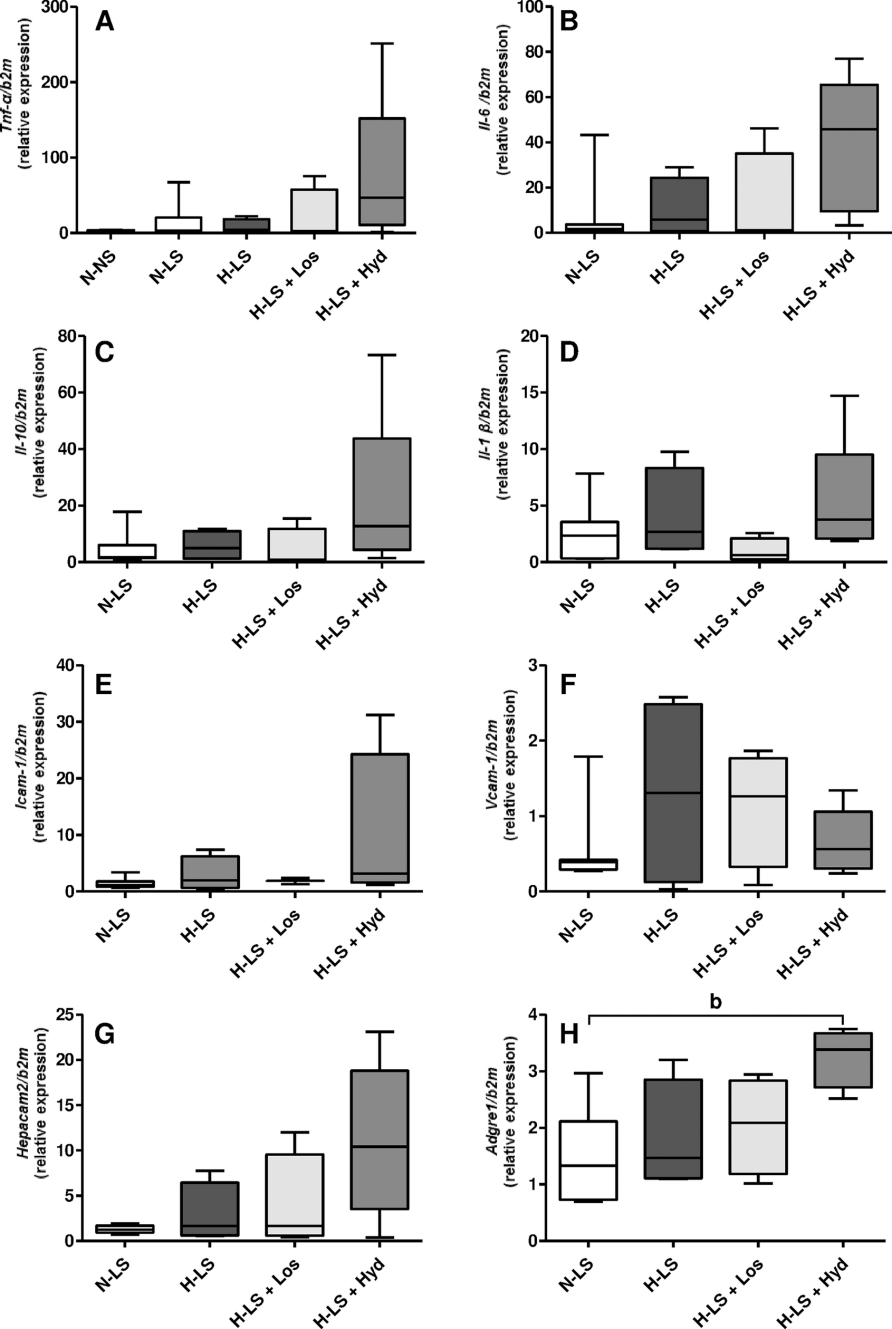
Gene expression (mRNA): A: TNF-α (*Tnf-α*); B: IL-6 (*Il6*); C: IL-10 (*Il10*); D: IL-1β (*Il1β*); E: ICAM-1 (*Icam1*); F: VCAM-1 (*Vcam1*); G: CD66 (*Hepacam2*); and H: F4/80 (Adgre1) from the mouse aortic arch. Data are expressed as relative mRNA units normalized to mouse β2M expression. The Mann Whitney test was used for comparisons between normotensive mice fed a normal-sodium (N-NS) diet and normotensive mice fed a low-sodium (N-LS) diet (A). ^b^ *P* < 0.05 hypertensive mice fed a low-sodium diet and treated with hydralazine (H-LS+Hyd) *vs* N-LS (H). The Kruskal Wallis test with Dunn’s post hoc test was applied for comparisons among the LS groups (A-H); n ≥ 4 mice per group.

Whether the vascular AT1 receptor and RAGE protein levels were related to their aortic arch gene expression levels was investigated. The quantitative RT-PCR analysis revealed that the AT1 receptor (*Agtr1*) and RAGE (*Ager*) mRNA contents did not differ among the groups (**Fig 6A and 6B**). To study the potential hyperlipidemic-induced inflammatory effect of the LS diet on vascular injury resulting from hyperlipidemia combined with hypertension, the gene expression levels of pro- and anti-inflammatory mediators were assessed. The TNF-α (*Tnf*), IL-6 (*Il6*), IL-10 (*Il10*), IL-1β (*Il1β*), ICAM-1 (*Icam1*), VCAM-1 (*Vcam1*) and CD66 (*Hepacam2*) gene expression levels in the aortic arch were not different among the groups (**Fig 7A–7G**). F4/80 (*Adgre1*) mRNA expression was enhanced by the Hyd treatment compared with the N-LS group (**Fig 7H**).

## Discussion

The current study showed that 2K1C hyperlipidemic H mice subjected to a long-term dietary sodium restriction (H-LS) had higher plasma TC and TG concentrations than did the H-NS mice. Although the LS diet lowered the BP, the H-LS mice developed vascular injury, as demonstrated by higher aortic lipid infiltration as well as CML and RAGE levels compared with those of the H-NS mice. With similar BP levels attained by the LS diet alone (N-LS), Los (H-LS+Los) effectively attenuated aortic damage, preventing arterial lipid infiltration along with increases in CML content and RAGE expression. Such effects suggest that the AT1 receptor influences the pathogenesis of atherosclerosis.

Genetic, pharmacological and environmental models of hypertension could favor pleiotropic effects through gene interactions and systemic dysfunctions, respectively. Such mechanisms could impair investigations into whether LS-induced BP reduction in H animals circumvents atherogenesis brought about by the LS-induced hyperlipidemia, the objective of the current study. Thus, mice with surgically induced hypertension were used to either avoid or reduce possible undesirable effects in our results that could be offered by other models of experimental hypertension.

It is worth noting that we investigated the early stages of atherogenesis since the fundamental difference between the diets is only in the sodium chloride concentration, which does not elicit the development of complex and advanced atherosclerotic plaques in LDLR KO mice.

A previous study from our laboratory showed that hypertensive non-obese patients following a chronic LS diet had increased plasma TG, glucose, insulin and arterial inflammatory marker concentrations, and a higher cholesterol content in chylomicron particles [25]. Additionally, Wistar rats chronically fed a LS diet had increased plasma TG, TC and NEFA concentrations due to a slower plasma TG removal rate [26]. We demonstrated the influence of LS on experimental atherogenesis in hyperlipidemic N LDLR KO and in apoE KO mice [6]. Arterial lipid infiltration was greater in LDLR KO mice on a LS diet. Interestingly, in this model, the plasma NEFA concentration was positively related to the arterial lipid infiltration area [6], suggesting that an insulin-resistance state was elicited by the dietary sodium restriction [30]. Additionally, it was reported that in apoE KO mice, dietary sodium intake was inversely related to plasma TC and ANG II concentrations as well as to atherosclerotic lesion area [23, 31]. Furthermore, LDLR KO mice fed a saturated fat-enriched diet containing different sodium concentrations (Na 0.01%, 0.1%, or 2% w/w) for 12 weeks had higher BP levels with high sodium intake and increased plasma cholesterol concentrations and atherosclerotic lesion areas with sodium restriction [24]. Increased plasma TC induced by a chronic LS diet could be involved in the onset and progression of experimental hypertension and atherosclerosis. Accordingly, hypercholesterolemia increases plasma angiotensinogen and angiotensin peptide (ANG II, III, IV, and 4–8) concentrations in LDLR KO mice [32] and vascular AT1 receptor levels in rabbits [33].

In the experimental model of simultaneous high-ANG II hypertension and dyslipidemia, increased BP is preserved by continuous RAAS activity and elevated circulating ANG II levels without hypervolemia [8].

As previously shown by ourselves [6, 25, 26] and others [20–24], the present study showed that dietary LS elicited increases in the plasma levels of TG and TC. Body weight and plasma volume were not different among the groups subjected to either the LS or NS diet; thus, these factors do not explain the modifications in the plasma lipid profile. Animals fed the LS diet had lower BP than mice receiving the NS diet, confirming the hypotensive effect of sodium restriction. In H mice, both Los and Hyd combined with dietary LS lowered BP more effectively than the LS diet alone; however, H-LS animals treated with Los exhibited less aortic lipid infiltration than did untreated H-LS animals (H-LS+Los *vs* H-LS).

Reductions in the plasma TG and NEFA levels of Los-treated mice were not attributed to reduced BP since the Hyd treatment also reduced BP without modifying the plasma TG and NEFA levels. Considering that ANG II causes insulin resistance and glucose intolerance [4], in the present study, the low plasma TG and NEFA concentrations in the Los-treated mice may have resulted from AT1 receptor antagonism, a condition that can ameliorate insulin sensitivity. Moreover, negative effects of ANG II on the arterial wall, such as the down-regulation of angiotensin-converting enzyme 2 (ACE2) expression [34], may have been prevented in the Los-treated mice (H-LS+Los). In the current study, reductions in plasma TG and NEFA levels, together with other anti-atherosclerotic mechanisms triggered by the AT1 receptor antagonism, may explain the lower lipid infiltration observed in the vascular wall of the Los-treated mice compared with the N-LS mice. The BP reduced by Los may have spared the aorta from lipid accumulation compared with that of H-LS animals not treated with Los. Nonetheless, the low lipid infiltration in the vascular wall after the Los treatment was not directly related to BP reduction since sodium restriction alone (N-LS) reduced BP to an extent similar to that attained by Los (**Table 2**), but with less protection against vascular lipid infiltration than that offered via Los (**Figs 2A, 2B and 3**). The plasma TC level in Los-treated mice was not different from those in H-LS+Hyd and N-LS mice (**Table 2**). Consequently, considering the effects of Los on plasma lipids and protection against vascular lipid infiltration, the major beneficial effect of Los on atherosclerosis can be ascribed to reductions in the plasma TG and NEFA concentrations. On the other hand, ANG II binding to the AT1 receptor and its cell signaling are important risk factors for accelerated atherosclerosis. Therefore, the benefits of RAAS blockade on atherogenesis, other than lowering plasma lipids, cannot be disregarded. Accordingly, injecting ANG II into hypercholesterolemic apoE KO mice enhances atherosclerosis with no significant effects on BP or plasma TC levels compared with placebo-treated mice. Peritoneal macrophages harvested after the intraperitoneal injection of ANG II exhibited cellular cholesterol biosynthesis increased by up to 90%. In contrast, peritoneal macrophages from mice treated with an angiotensin-converting enzyme inhibitor exhibited up to a 70% reduction in cholesterol biosynthesis [35]. Thoracic aorta atherosclerotic lesion area was greater in ANG II-induced hypertensive apoE KO mice than in norepinephrine-induced hypertensive animals [36]. In addition, ANG II increased NADPH oxidase activation in vascular cells [37] and mitochondrial reactive oxygen species (ROS) production [38]. In the current investigation, the higher CML content in the aorta of mice on the LS diet (N-LS and H-LS) suggests greater ANG II-induced lipid peroxidation than that in animals on the LS diet and treated with an antihypertensive drug (Los or Hyd). Hyd efficiently traps carbonyl compounds that are produced during oxidative stress, lipid peroxidation and other deleterious cellular processes [39]. In the present study, the Hyd carbonyl-sequestering property spared the aortic arch from increased CML content compared with that of the N-LS group (**Figs 4 and 5C**), and the Los antioxidant function [40] protected mice from vascular lipid infiltration (**Figs 2A, 2B and 3**) and injury (**Figs 4 and 5C–5F**), wherein Los showed more efficient vascular protection. The higher plasma TC and TG concentrations in the H-LS group than in the H-NS group may have led to increased lipid peroxidation, which is an important source of CML (**Fig 5C and 5D**) biogenesis [41]. It is worth noting that the CML and RAGE levels were similar in the histological aortic sites we investigated and that the experimental groups with higher CML and RAGE levels also showed increased vascular lipid infiltration. The accumulation of RAGE ligands upregulates RAGE expression in a positive-feedback loop in several cell types, which may explain the similar aortic CML and RAGE levels observed in the experimental groups. In the present report, the RAGE mRNA level in the aortic arch did not differ among the experimental groups. Perhaps the differences in RAGE expression demonstrated by the RT-PCR and immunofluorescence assays can be attributed to the fact that this gene expresses several splice variants in mice, dogs and humans [42, 43].

Since ANG II-induced oxidative stress decreases AT1 receptor expression [44], it could be speculated that, compared with H-NS mice, the lower AT1 receptor expression in the H-LS mice was due to greater oxidative stress (**Fig 5A**). In this sense, induced renovascular hypertension associated with severe dietary sodium restriction (H-LS group) may favor more intense RAAS activity than that in animals with renovascular hypertension (H-NS) alone. However, the lower aortic arch AT1 receptor expression in H-LS mice than in H-NS mice was not related to the AT1 receptor mRNA level. The possibility that AT1 receptor expression is modulated by variations related to hemodynamic factors, such shear stress, oscillating flow and inherent vessel wall properties in different aortic arch segments, cannot be ruled out. Accordingly, it has been shown that site-specific atherosclerosis is generally favored by the differential expression of genes related to cell adhesion, proliferation, differentiation, cell death, lipid metabolism and immune responses [28]. In this sense, in the present study, the lower AT1 receptor expression in H-LS mice was present only in aortic arch segments I-II, while the AT1 receptor mRNA content was determined in the whole aortic arch, i.e., in all aortic segments together (I-II and III-IV). On the other hand, the AT1 receptor protein level was markedly increased in the Hyd-treated mice compared with the H-LS+Los mice. Although Hyd has carbonyl-scavenger and antiatherogenic properties, but only moderate antioxidant activity [45], some investigations have stressed Hyd as an efficient ROS scavenger and an inhibitor of superoxide generation [46]. Thus, in the Hyd-treated mice, these antioxidant properties may have disturbed the negative-feedback control of AT1 receptor expression, which is mediated by ANG II-induced ROS generation, thereby favoring a high AT1 receptor expression level (**Fig 5A**).

Hyd elicited intense inflammatory F4/80 (*Adgre1*) gene expression compared with that observed in the N-LS group. Some investigations have shown that Hyd causes immunotoxic side-effects and various allergic reactions, including hypersensitivity reactions and drug-induced auto-immune responses in humans [47] and animals [48] that may boost F4/80 (*Adgre1*) gene expression, as observed in the Hyd-treated mice of the present study (**Fig 7H**).

These results indicate that ANG II is pro-atherogenic mainly due to its humoral effect and not merely to its hypertensive mechanical effect [36], which highlights the influences of AT1 receptor signaling and RAAS blockade on atherosclerosis other than changes in plasma lipid levels. Although BP was higher in the H-LS group, arterial lipid accumulation and plasma TC, TG and NEFA concentrations in H-LS mice did not differ from those in N-LS mice. Thus, it is difficult to draw conclusions on the effective influence of BP on arterial lipid infiltration.

As expected, in this study, differences between the right and left renal masses [8] corroborated the establishment of renal ischemia. There were no changes in the renal weights of N animals. The 24-hour UNa excretion reflected the sodium intake of the animals. ANG II is known to stimulate erythropoiesis by increasing the synthesis of erythropoietin and acting as a growth factor for myeloid erythrocytic progenitor cells. These mechanisms may explain our finding of decreased hematocrit in Los-treated mice.

The current study shows that despite its antihypertensive effect, the LS diet led to higher plasma TC and TG concentrations (N-LS *vs* N-NS; H-LS *vs* H-NS), greater aortic lipid accumulation, enhanced vascular CML content and RAGE expression and decreased AT1 receptor expression (H-LS *vs* H-NS mice) than did the NS diet. It could be surmised that the greater lipid accumulation along with the increased CML content and RAGE expression in the vascular wall of H-LS mice might be due to increased RAAS activity resulting from the LS diet combined with renovascular hypertension; nonetheless, the present results cannot definitively validate this point. When considering BP and plasma TC, TG and NEFA concentrations, the univariate analysis showed that plasma TG and NEFA concentrations are related to arterial lipid infiltration area in the experimental groups, which may be attributed to a state of insulin resistance, as previously reported [30].

In our report, aortic lipid infiltration and the CML and RAGE contents were greater in H-LS mice than in H-NS mice, despite the former presenting lower BP than the latter. However, the present study should not overlook that the greater lipid infiltration into the vascular wall and the higher CML and RAGE levels derived from chronic sodium restriction were observed in a unique dyslipidemic murine model. Therefore, the present results cannot contradict the goal of worldwide reduction in salt intake, as recommended by the American Medical Association, the American Heart Association, the American Society of Hypertension and the World Health Organization. Taken together, these data support an important role of ANG II in vascular lipid accumulation, CML and RAGE levels in the context of experimental dietary LS and systemic RAAS activation.

The present investigation shows that severe chronic sodium restriction worsens arterial lipid infiltration due to dyslipidemia and increases the vascular CML and RAGE levels in a murine model of simultaneous hyperlipidemia and ANG II-dependent hypertension. Vascular protection via AT1 receptor blockade may be attained by BP-independent mechanisms, such as reducing the plasma TG and NEFA concentrations, since chronic dietary sodium restriction alone also reduced BP but was less effective than Los in preventing arterial lipid infiltration along with CML content and RAGE expression. Accordingly, adequate RAAS modulation combined with an improved plasma lipoprotein profile may contribute to cardiovascular disease prevention and treatment in the context of dyslipidemia and hypertension. Despite reducing BP, the LS diet in H mice caused arterial lipid infiltration due to increased plasma TC and TG levels. Los and Hyd reduced BP in H-LS mice, but Los was likely more effective than Hyd for also reducing plasma TG and NEFA levels and preventing arterial injury.

Thus, the novelty of the current study lies in the observation that the pleiotropic effect of chronic and severe sodium restriction elicited aortic damage even with reduced BP in a murine model of simultaneous hypertension and hyperlipidemia. In this model, such negative effects on the arterial wall were limited by the protective role of AT1 receptor antagonism, which demonstrates the influence of ANG II in atherogenesis induced by a severely LS diet. Due to the pleiotropic effects of long-term sodium restriction, the precise mechanism by which a LS diet favors atherogenesis is not certain. However, even without investigating cellular mechanisms, which are the subject of ongoing studies in our laboratory, we have provided *in vivo* evidence that minimizes possible study design limitations affecting our findings.
